# Supplementary figures and images for: TLR4 promoter rs1927914 variant contributes to the susceptibility of esophageal squamous cell carcinoma in the Chinese population
Source: PeerJ. 2021 Feb 1;9:e10754. doi: 10.7717/peerj.10754 (PMC7860108; doi:10.7717/peerj.10754)

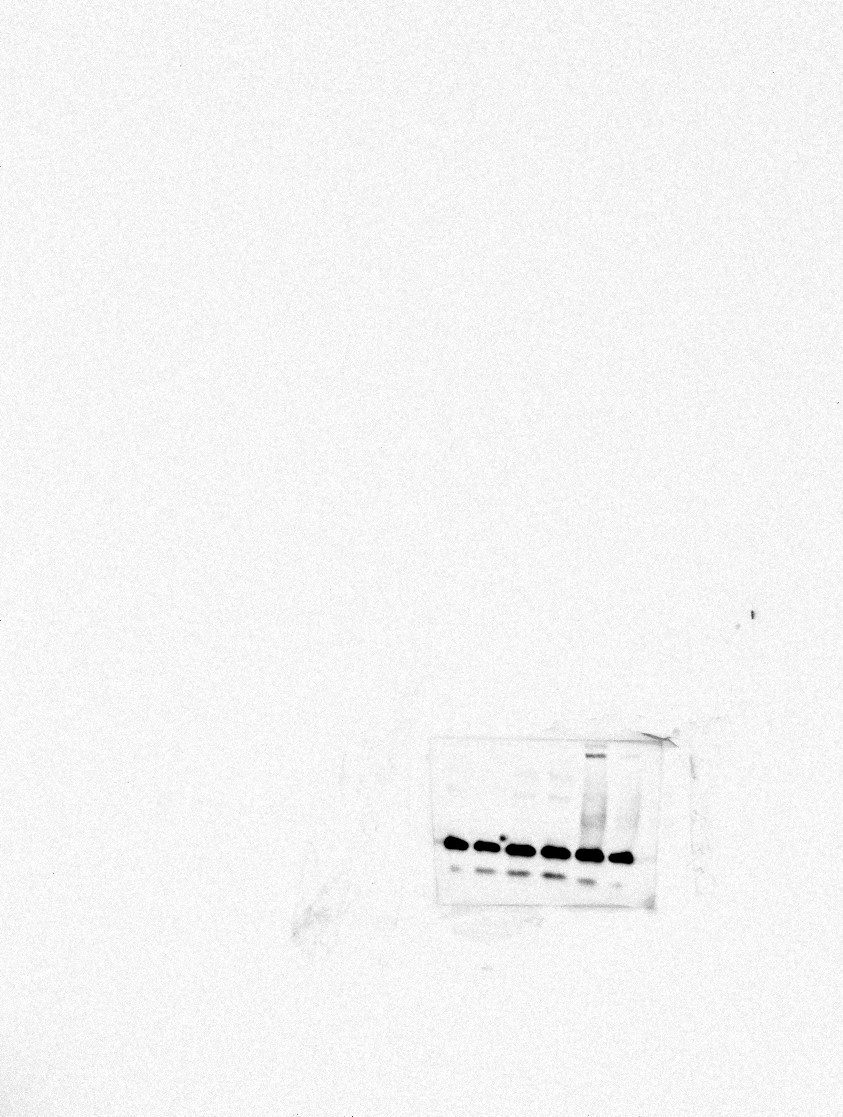

Supplement: Supplemental Information 1 [file peerj-09-10754-s001.jpg]
